# Low temperature decreased insecticidal protein contents of cotton and its physiological mechanism

**DOI:** 10.3389/fpls.2022.1082926

**Published:** 2023-01-24

**Authors:** Yuan Chen, Zhenyu Liu, Yuyang Dai, Ye Yue, Yuting Liu, Hanjia Li, Run He, Xiang Zhang, Dehua Chen

**Affiliations:** Jiangsu Key Laboratory of Crop Genetics and Physiology, Co-Innovation Center for Modern Production Technology of Grain Crops, Yangzhou University, Yangzhou, Jiangsu, China

**Keywords:** Bt cotton, low temperature, insecticidal protein, nitrogen metabolism, square

## Abstract

Low temperature delayed cotton growth, increased abscission of reproductive organs, and seriously reduced quality and yield. Moreover, failed or unstable performance of insecticidal resistance due to adverse environments have been reported. In order to study the impact of low temperature on the insecticidal protein contents at square stage in *Bacillus Thuringenesis* (Bt) transgenic cotton, different temperature regimes and durations were imposed on two Bt cotton cultivars, Sikang1 (the conventional cultivar, SK1) and Sikang3 (the hybrid cultivar, SK3). Low temperature stress exhibited a significant inhibitory effect on insecticidal protein expression in squares and leaves of Bt transgenic cotton plants, with insecticidal protein contents decreased up to 30% with decreasing temperature. In addition, the threshold temperature resulting in significant reduction of insecticidal protein contents symbolized a rising trend as stress duration extended, together with a greater reduction observed. Thus, at square stage, the detrimental influence of low temperature on Bt protein contents was closely related to the low temperature level and duration. The square Bt protein content was positively correlated with leaf Bt protein content, but was more sensitive to low temperature. Across the whole treatment duration in both years, square Bt protein level was significantly negatively correlated with malondialdehyde (MDA) contents, as well as the activities of catalase (CAT) and superoxide dismutase (SOD), indicating the negative effect of cold induced oxidative stress on Bt protein contents. The reduced Bt protein contents under low temperature were also related to altered N metabolism. Glutamate oxaloacetate transaminase (GOT) and glutamate pyruvate transaminase (GPT) activities, as well as soluble protein contents in squares reduced, and greater reduction was recorded with decreasing temperature. In contrast, the free amino acid contents, and peptidase and protease activities increased, and greater elevation was noted with decreasing temperature. These results suggested in Bt cotton production, it is necessary to be alert to low temperature disasters that may last for more than 24 hours and lower than 15-17°C during the square stage, which may lead to reduced insecticidal resistance causing serious economic losses.

## 1 Introduction

Since the large-scale promotion and application of transgenic *Bacillus Thuringenesis* (Bt) insect-resistant cotton in 1997, the area of Bt cotton has accounted for more than 90% of the cotton planting area in China (Qiao et al., 2017). Bt cotton can effectively reduce the harm of cotton bollworm and other pests and thus reduce the dosage of pesticides (Huang et al., 2002; Deguine et al., 2008). The application of Bt cotton not only saves the labor and cost of cotton planting, but also reduces the pollution of pesticides to the environment, which has good economic and ecological benefits (Wu et al., 2008; Lu et al., 2012; Han et al., 2014; Ali et al., 2016; Qiao et al., 2017).

However, it has been widely agreed that the insect resistance of Bt cotton is unstable (Dong and Li, 2007). There were temporal and spatial differences in the expression of insect resistance in transgenic Bt cotton. In terms of timing, the expression of insect resistance decreased with the growth stage; the insect resistance is strong at seedling stage but weak at boll stage; the resistance to the 2nd generation of cotton bollworm is higher, and the resistance to the 3rd and 4th generations of cotton bollworm decrease significantly (Cui and Xia, 1999; Adamczyk and Meredith, 2004; Torres et al., 2006). Spatially, the insect resistance of different organs is different, and the most important feature is that the insect resistance of reproductive organs is significantly lower than that of leaves (Huang et al., 2010). A variety of environmental factors affect the expression of insecticidal protein in Bt cotton. The growth of Bt cotton plants under moderate water-deficit conditions exhibited reduced Bt levels in leaves, flowers and bolls (Martins et al., 2008). Drought and waterlogging significantly reduce the content of Bt protein in different organs of cotton plant, and drought has a greater impact on the expression of Bt protein (Zhang et al., 2017). Salinity stress significantly reduced the content of Bt insecticidal protein and the insect resistance of Bt cotton (Jiang et al., 2005; Luo et al., 2017; Wang et al., 2018). The decreased insecticidal efficacy under waterlogging or the combined stress of salinity and waterlogging was mainly related to the declined levels of Bt protein (Luo et al., 2008). High temperature makes the silencing time of Bt gene earlier, and the insecticidal protein expression reduce in advance accordingly, resulting in a sharp decrease in insecticidal protein content (Chen et al., 2012; Heng et al., 2016). Extreme air humidity would also lead to a significant decrease in insecticidal protein content, and further aggravate the impact of temperature stress on insecticidal protein content (Chen et al., 2012). When the concentration of insecticidal protein drops below the lethal level of 1.9g, cotton may be attacked by cotton bollworm again (Liu et al., 2019). Therefore, exploring the effect of adverse environment on the expression of insecticidal protein in Bt cotton has become an important part of insect resistance.

Cotton is native to the tropics and subtropics, and is extremely sensitive to low temperature. Low temperature impaired the seed germination (Xia et al., 2022), caused damage to the photosynthetic apparatus (Snider et al., 2022), increased abscission of reproductive organs (Shu et al., 2022), delayed cotton growth, and seriously reduced quality and yield (Zhou et al., 2015). The low temperature of 15-20°C often occurs in China’s cotton planting areas from mid June to mid August every year, and lasts for 5-7 days. At present, China’s cotton producing areas are gradually shifting to Xinjiang, so low-temperature stress occurs more frequently in cotton production, which is manifested as low temperature in spring and the rapid cooling in autumn. The cotton flowering and bolling period last from late July to early August, which is prone to low temperature stress, especially the early frost would cause serious yield decrease. At the end of July 2001, there were four consecutive days of low temperature and rainy weather in northern Xinjiang, resulting in serious damage to 266,700 hectares of cotton, a yield loss of 40% - 50%, and an economic reduction of up to 1 billion yuan. In August 2004, cotton in northern Xinjiang experienced low temperature stress with an average temperature lower than 19°C and a minimum temperature of 8.4°C for three consecutive days. After 1-2 weeks, leave senescence and boll abscission was observed, flower buds fall off, and the yield decreases by 30% - 40% (Lai et al., 2008).

Chen et al. (2012) also confirmed that the effect of low temperature stress on the expression of Bt insecticidal protein is greater than that of high temperature. In the bolling stage, when Bt cotton was under low temperature and flooding stress, the expression of insecticidal protein decreased by 53% (Zhou et al., 2015). Although previous research reported the changes of insect resistance of Bt cotton under low temperature, there is a lack of systematic research on the effects of low temperature level and duration on the expression of Bt insecticidal protein. Previous studies mainly focused on the effect of low temperature on the expression of insecticidal protein in leaves, while the effects on the reproductive organs, which was preferred by cotton bollworm, were rarely studied. The existing results showed that the content of insecticidal protein in reproductive organs such as squares and bolls were significantly lower than that in leaves. Squares and bolls are not only the first choice of cotton bollworm, but also the yield object of cotton cultivation. The expression of their insecticidal proteins can better reflect the insect resistance of Bt cotton. To this end, aiming at the problem that low temperature affects insect resistance in cotton production practice, the present study aims to explore: (1) the effects of low temperature level and stress duration on the expression of Bt insecticidal protein in the reproductive organs of Bt cotton; (2) The underlying mechanism that low temperature affects the expression of Bt insecticidal protein in reproductive organs.

## 2 Materials and methods

### 2.1 Experimental materials and design

The study was conducted using two Bt transgenic cotton cultivars, ‘Sikang3’ (hybrid) and ‘Sikang1’ (conventional), at Yangzhou University, Yangzhou, China (32 ° 30’N, 119 ° 25’E) during 2020-2021 cotton growing season. Seeds were sowed in greenhouse on April 15th in 2020 and April 18th in 2021, and the seedlings were transplanted to the pots (50-cm height, 40-cm diameter, 62.8-L volume) at 35 days after sowing. The pots were filled with 20 kg sandy loam soil (Typic fluvaquents, Entisols), which contained 18.8 g kg^-1^ organic matter and available N-P-K at 135.2, 22.8 and 80.9 mg kg^-1^ respectively. Plants were watered thoroughly on a daily basis. On the day of transplanting (May 17th), 1.5 g N as urea, 0.7 g P as single superphosphate and 2.6g as KCl were mixed into the soil of each pot, and one seedling was transplanted in each pot. At 46 days after transplanting, 1.60 g N as urea, 0.7 g P as single superphosphate and 2.6g as KCl were top-dressed into each pot. At 68 days after transplanting, 2.0g N as urea were top-dressed into each pot.

The experiments were performed utilizing a completely randomized design with six replications that consisted of six temperature regimes (optimum temperature: 27°C and low temperature: 15°C, 16°C, 17°C, 18°C, 19°C) in 2020 and five temperature regimes (optimum temperature: 27°C and low temperature: 15°C, 16°C, 17°C, 18°C) in 2021. At peak square stage (Jun 24th), the square appearance on third to fifth fruiting branches were recorded and the temperature treatments were initiated at fifteen days after square appearance by transporting pots to environmentally controlled rooms with different temperature settings (14 h d^–1^ photoperiod at a photon flux density of 200 μmol m^–2^ s^–1^; and 70% relative humidity). Labeled squares and the 4th uppermost fully-extended leaves were collected at 12h, 24h, and 48h after treatments and stored at -80°C for later measurements.

### 2.2 Data collection

Malondialdehyde (MDA, the final product of lipid peroxidation) was measured to test the level of lipid peroxidation. A 0.8 g leaf sample was homogenized in 10 mL of 10% trichloroacetic acid (TCA) and centrifuged for 10 min at 4,000 g. A 3-mL portion of the supernatant was mixed with 3 mL of 10% TCA containing 0.6% 2,4,6-tribromoanisole (TBA). The mixture was incubated in a 100 °C water bath for 15 min. Samples were centrifuged for 10 min at 4,000 g after a 5-min ice bath. The absorbance for the supernatant was determined spectrophotometrically (DU Series 500, Beckman Instruments Inc., CA) at 532 nm, 600 nm, and 450 nm to calculate the MDA content. Lipid peroxidation was expressed as the MDA concentration in µM g^-1^ FW. Superoxide dismutase and catalase was assayed with the test kits (Keming, Suzhou) with 0.2 g leave samples.

As described by Chen et al., (Chen et al., 1997), *Cry1Ac* endotoxin content was analyzed by immunological analysis ELISA. We analyzed the the total free amino acid content by ninhydrin assay according to Yemm et al., (Yemm et al., 1955). The total soluble protein concentration was determined by the Coomassie Blue dye-binding assay of Bradford (1976). Samples (0.2 g) were homogenized in 5 mL 0.05 mM Tris-HCl butter (pH 7.2) and the homogenate was centrifuged at 26,100 g at 4°C for 10 min. The supernatant was used for the analysis of GOT and GPT activities, following the procedure described by Tonhazy et al. (1950). Samples (0.8 g) were homogenized at 4°C in 1 ml of β-mercaptoethanol extraction buffer (a mixture of ethylene glycol, sucrose, and phenylmethylsulfonyl fluoride pH 6.8). The supernatant was collected to estimate the square protease activity (Vance et al., 1979). For peptidase activity measurement, samples (0.5 g) were homogenized in 8 ml Tris-HCl buffer (4 mM DTT, 4 mM EDTA, 1% PVP, pH 7.5) at 4°C and measured for the peptidase activity based on Setlow (1975).

### 2.3 Statistical analysis

The statistical significance between means were determined by analysis of variance (ANOVA) and multiple mean comparisons were analyzed by LSD (α=0.05) in SAS 9.4 (SAS Institute, NC). The correlation analysis was conducted with Pearson’s correlation analysis.

## 3 Results

### 3.1 Low temperature effect on the insecticidal protein content of cotton plants

**Figure1:**
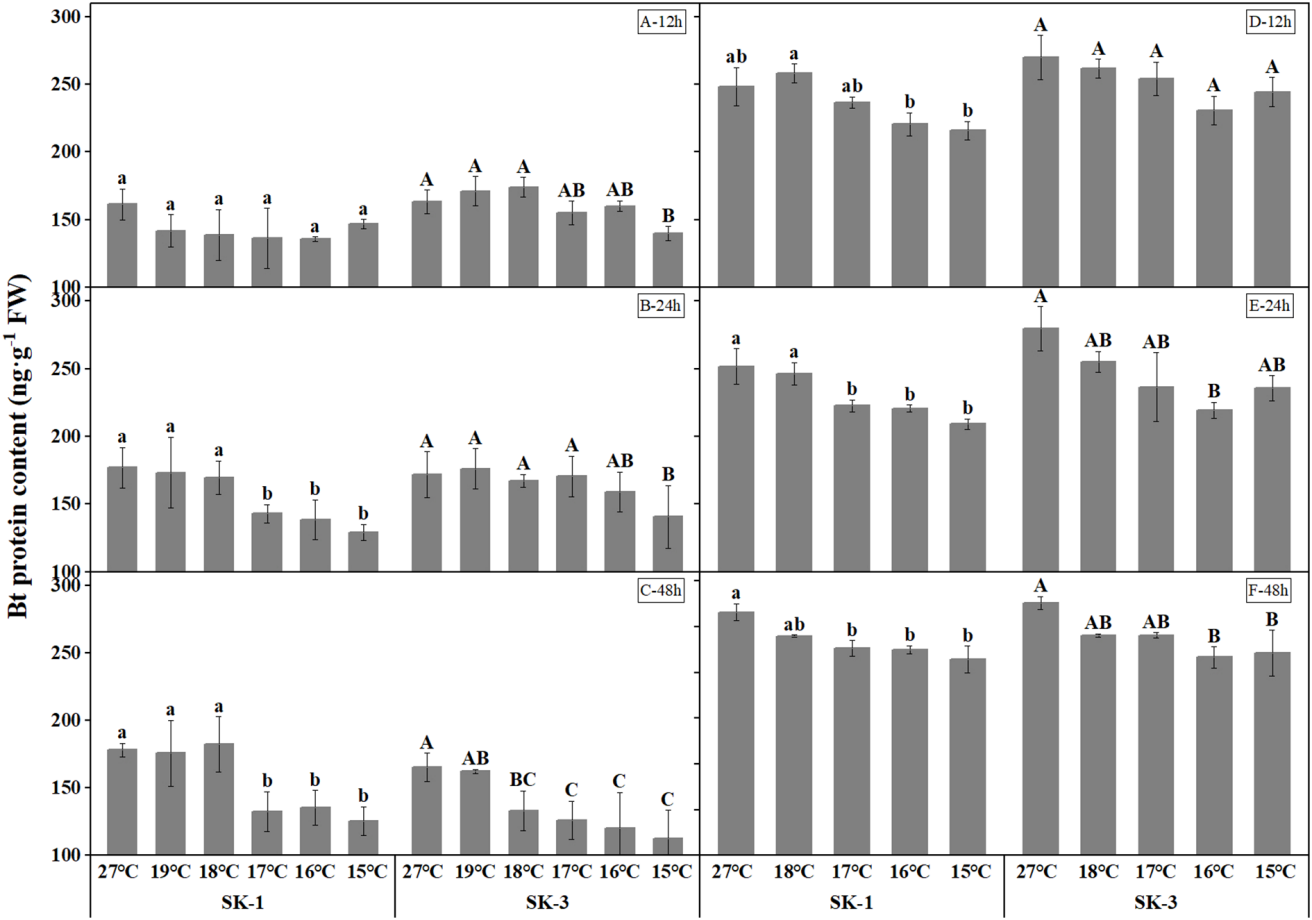
Effect of low temperature stress on Bt insecticidal protein content in cotton squares in 2020 (**A-C**: 12h, 24h, 48h stress) and 2021 (**D-F**: 12h, 24h, 48h stress). FW represents fresh weight. Different lowercase letters indicate the significant differences between the treatments of the variety SK1, and different capital letters indicate the significant differences between the treatments of the variety SK3 (P<0.05).

Compared with the control (27°C), the content of insecticidal protein in squares decreased under low temperature treatment, and with the decreasing temperature, its content showed a decreasing trend (**Figure 1**). In 2020, after 12h of low temperature treatment, compared to the optimum temperature (27°C), the Bt protein content reduced significantly at 15°C in SK3. After 24h of low temperature treatment, the content of insecticidal protein in SK1 decreased significantly at 15°C, 16°C and 17°C, and declined significantly at 15°C in SK3. After 48 hours of low temperature treatment, the content of insecticidal protein in SK1 decreased significantly at 15°C, 16°C and 17°C, and the content of insecticidal protein in SK3 reduced significantly at 15°C, 16°C, 17°C, 18°C. The above results show that the critical low temperature that causes the significant reduction of insecticidal protein content in SK1 was 17°C after 24h and 48h stress, and in SK3 the threshold temperature was 15°C after 12h and 24h stress, and 17°C after 48h of low temperature treatment. In 2021, similar trend was observed for Bt protein content in squares, with the threshold temperature as 16°C, 17°C, 17°C respectively after 12h, 24h, and 48h of low temperature stress in SK1 and 16°C after 12h, 24h, and 48h of low temperature stress in SK3. According to above results, the critical low temperature that causes the significant reduction of insecticidal protein content in squares increased with the prolongation of low temperature duration.

In 2020, after low temperature treatment for 12h, the Bt protein content was decreased by 9.01%, 15.84%, 15.55%, 13.86%, 12.03% in SK1 and 14.40%, 1.96%, 4.97%, -6.64%, -4.79% in SK3 at 15°C, 16°C, 17°C, 18°C, 19°C. After 24h of low temperature treatment, the Bt protein content was decreased by 26.99%, 21.62%, 19.15%, 4.10%, 2.05% in SK1 and 18.15%, 7.45%, 0.74%, 2.57%, -2.62% in SK3 at 15°C, 16°C, 17°C, 18°C, 19°C. After 48h of low temperature treatment, the Bt protein content was decreased by 29.49%, 24.08%, 25.63%, -2.32%, 1.35% in SK1 and 31.91%, 27.30%, 23.86%, 19.42%, 1.89% in SK3 at 15°C, 16°C, 17°C, 18°C, 19°C. Similar trend was detected in 2021. It can be seen from the above data that prolonging the low temperature stress treatment duration not only increased the threshold temperature, but also further reduced the content of insecticidal protein compared with the control.

**Figure 2:**
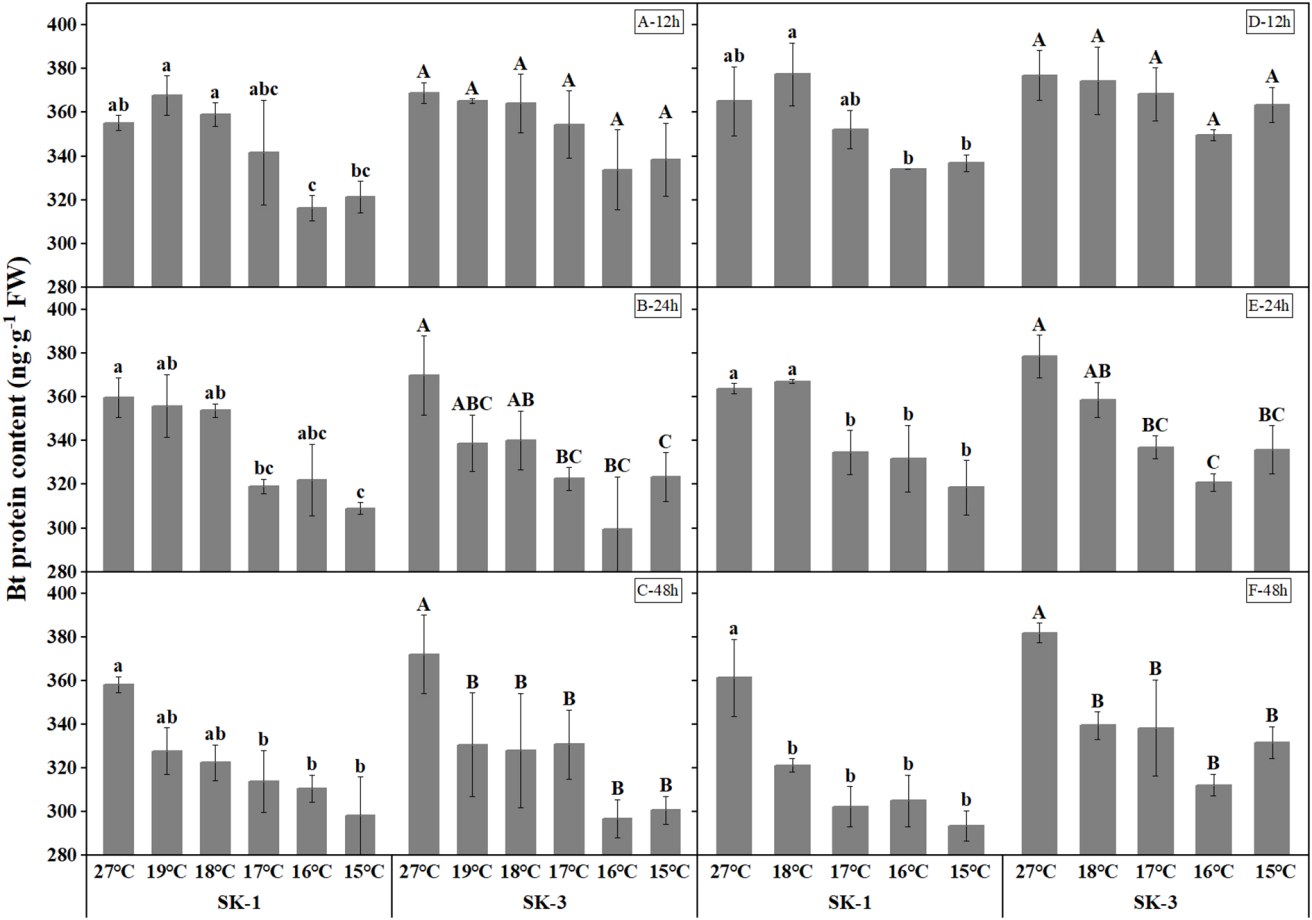
Effect of low temperature stress on Bt insecticidal protein content in cotton leaves in 2020 (**A-C**: 12h, 24h, 48h stress) and 2021 (**D-F**: 12h, 24h, 48h stress). FW represents fresh weight.Different lowercase letters indicate the significant differences between the treatments of the variety SK1, and different capital letters indicate the significant differences between the treatments of the variety SK3 (P<0.05).

Similar trend was observed in insecticidal protein content in leaves to that in squares, insecticidal protein content in leaves reduced with decreasing temperature (**Figure 2**). Also, as the low temperature stress treatment duration increased, the threshold temperature enhanced, and greater decrease of insecticidal protein was noted. In 2020, after 48h of low temperature treatment, the Bt protein content was decreased by 16.80%, 13.33%, 12.37%, 9.97%, 8.50% in SK1 and 19.20%, 20.24%, 11.08%, 11.83%, 11.13% in SK3 at 15°C, 16°C, 17°C, 18°C, 19°C.

**Figure 3:**
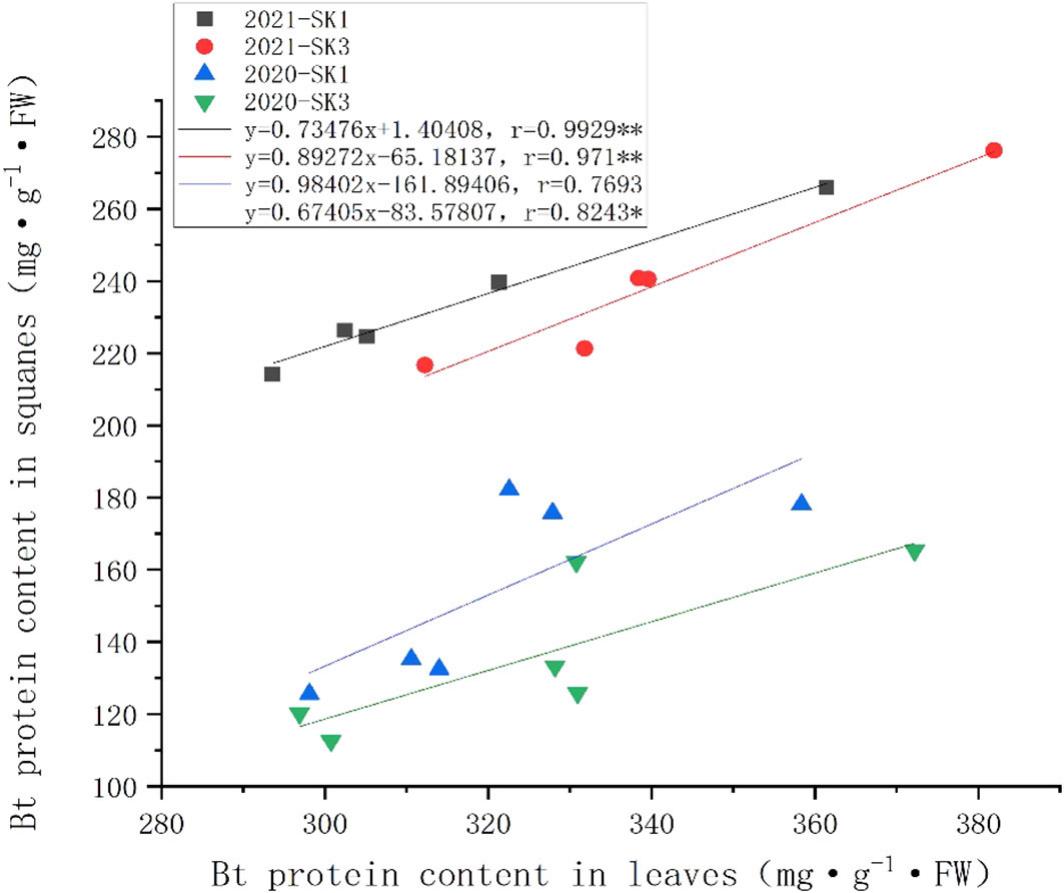
Correlations between Bt insecticidal protein content in leaves and squares after 48 h low temperature stress in 2020 and 2021. Asterisks indicate significance at 0.05 (*) and 0.01 (**) level.

The relationship between the insecticidal content between leaves and squares was presented in **Figure 3**. Across the whole treatment duration, there was a significant positive correlation between square Bt toxin level and leave Bt toxin level. Compared to the changes in square under low temperature, the insecticidal protein content in leaves showed less reduction, indicating squares was more sensitive to cold stress in terms of insecticidal protein content.

### 3.2 Low temperature effect on antioxidant activity of cotton plants

**Figure 4:**
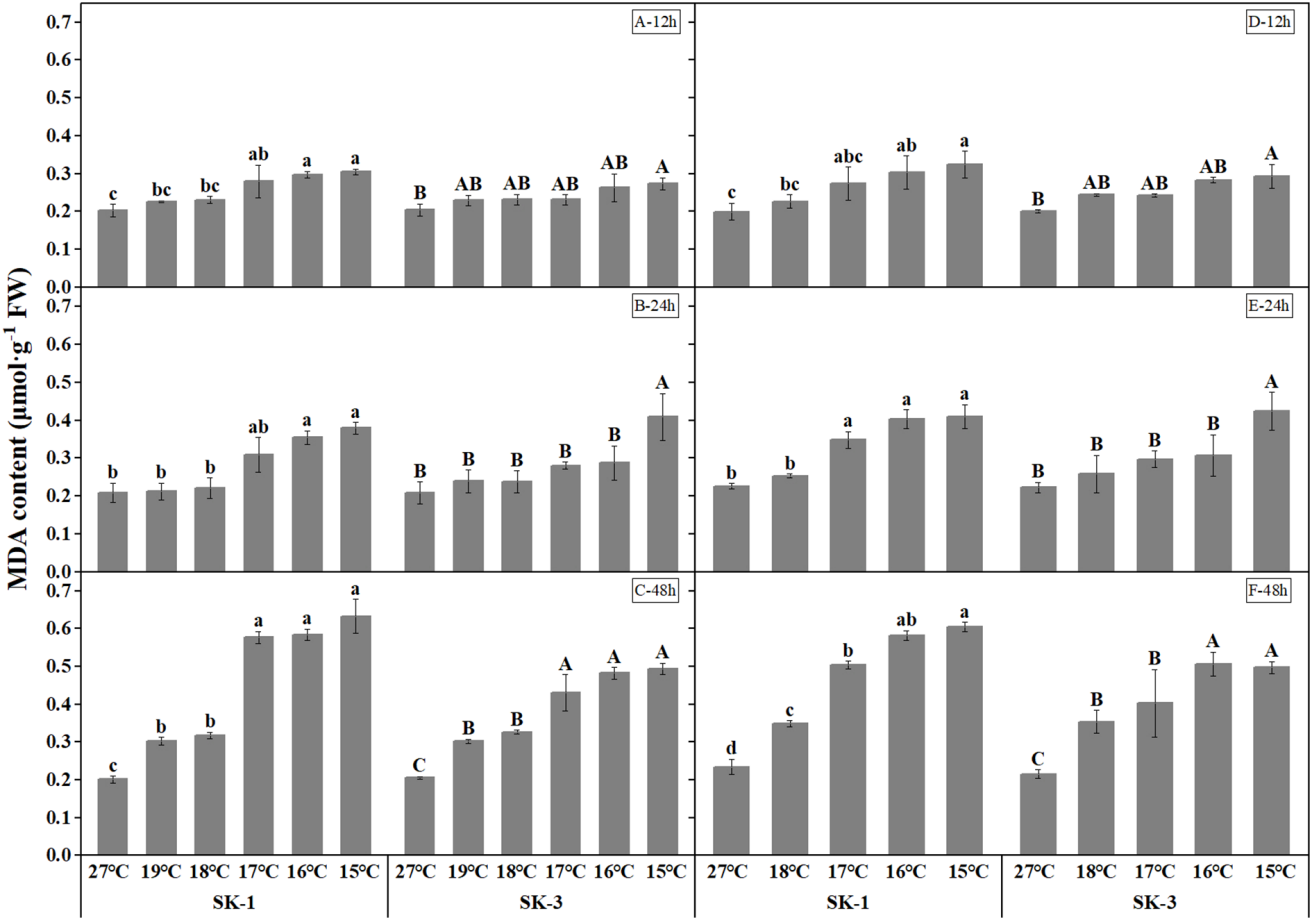
Effect of low temperature stress on MDA content in cotton squares in 2020 (**A-C**: 12h, 24h, 48h stress) and 2021 (**D-F**: 12h, 24h, 48h stress). FW represents fresh weight.Different lowercase letters indicate the significant differences between the treatments of the variety SK1, and different capital letters indicate the significant differences between the treatments of the variety SK3 (P<0.05).

MDA, a decomposition product of polyunsaturated fatty acid hydroperoxides that is widely used to quantify lipid peroxidation, was measured in our study (**Figure 4**). Greater MDA content in response to a lower temperature regime and a longer low temperature duration was noted, suggesting a detrimental effect of low temperature on membrane integrity. A significant temperature regime effect was observed starting at 17°C, 17°C, 19°C in SK1, and 16°C, 16°C, 17°C in SK3 in 2020, and at 16°C, 17°C, 17°C in SK1, and 16°C, 16°C, 17°C in SK3 in 2021.

**Figure 5:**
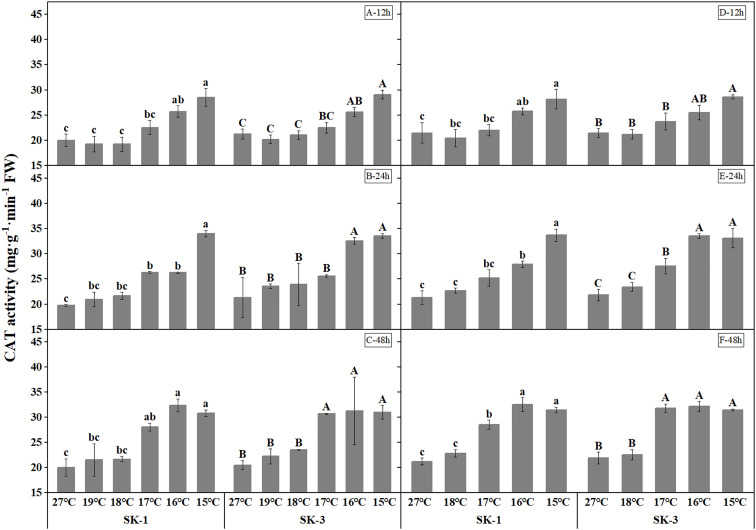
Effect of low temperature stress on CAT activity in cotton squares in 2020 (**A-C**: 12h, 24h, 48h stress) and 2021 (**D-F**: 12h, 24h, 48h stress). FW represents fresh weight. Different lowercase letters indicate the significant differences between the treatments of the variety SK1, and different capital letters indicate the significant differences between the treatments of the variety SK3 (P<0.05).

**Figure 6:**
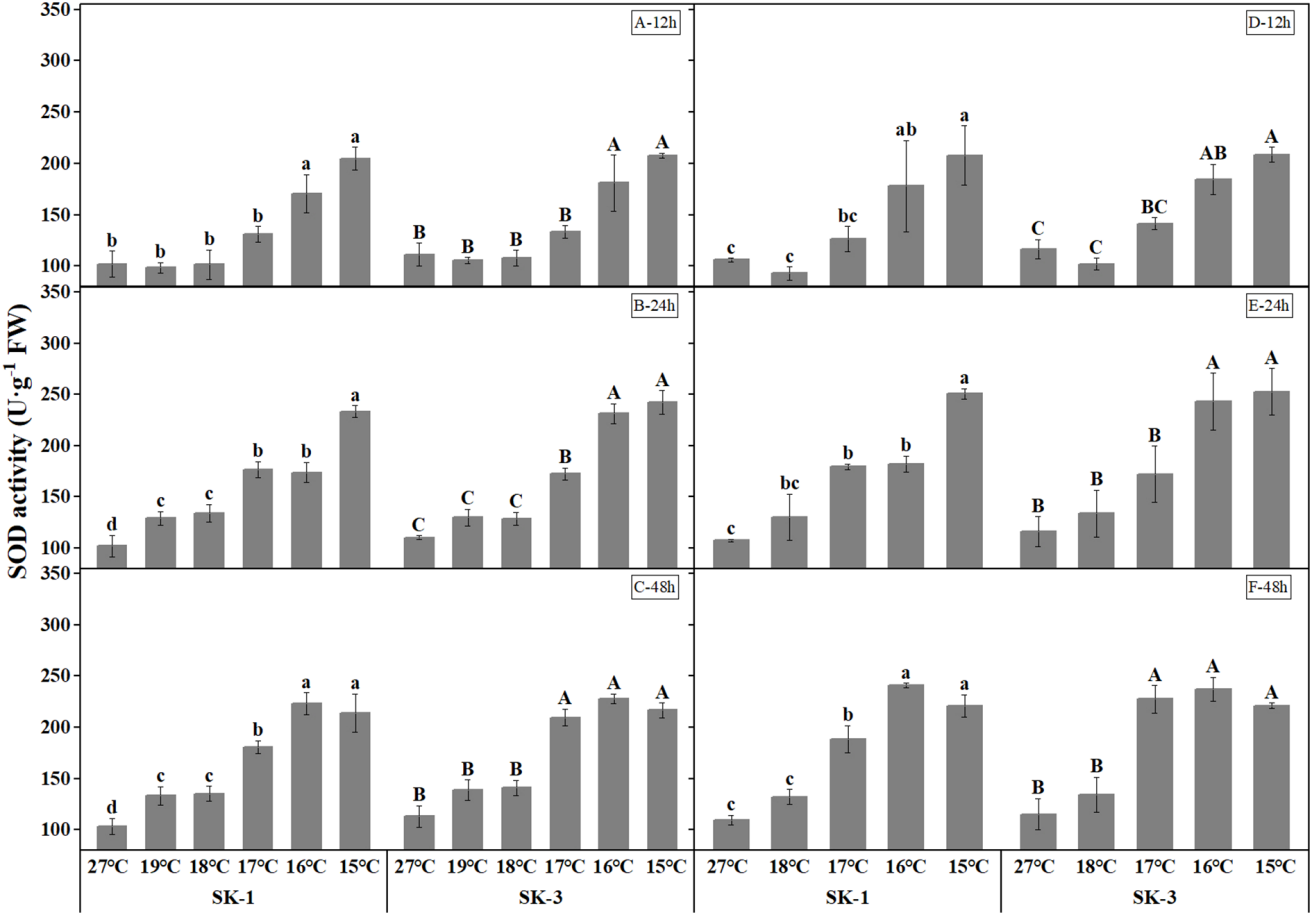
Effect of low temperature stress on SOD activity in cotton squares in 2020 (**A-C**: 12h, 24h, 48h stress) and 2021 (**D-F**: 12h, 24h, 48h stress). FW represents fresh weight. Different lowercase letters indicate the significant differences between the treatments of the variety SK1, and different capital letters indicate the significant differences between the treatments of the variety SK3 (P<0.05).

Cotton plants exposed to low temperature exhibited higher catalase and superoxide dismutase activities, which indicates a higher antioxidant activity under stress (**Figures 5**, **6**). As the low temperature duration extended, a lower temperature threshold causing significant increase in enzyme activities was recorded. In 2020, a significant temperature regime effect started at 16°C, 17°C, 17°C in SK1, and 16°C, 16°C, 17°C in SK3 for the CAT enzyme activity, and for SOD enzyme activity, the effect commenced at 16°C, 19°C, 19°C in SK1, and 16°C, 17°C, 17°C in SK3. Thus, according to above data, the low temperature stress increased CAT and SOD activities, as well as MDA content in stressed cotton plants. And the effect of stress strengthened with lower temperature and longer stress duration.

Across the whole treatment duration in each year, there was a significant negative correlation between MDA contents and square Bt toxin level, with values of -0.9669** for SK1 and -0.9323** after 48h stress for SK3 in 2020, and -0.9606** for SK1 and -0.9858** for SK3 in 2021. In addition, square Bt toxin level was significantly negatively correlated with activities of CAT and SOD (**Table 1**), indicating the negative effect of cold induced oxidant stress on Bt protein contents.

**Table 1:** Correlations between Bt insecticidal protein content and antioxidant ability.

Bt insecticidal protein content		SK1			SK3		
		MDA	CAT	SOD	MDA	CAT	SOD
2020	12h	-0.5506	-0.1592	-0.1854	-0.631	-0.8795*	-0.8501*
	24h	-0.9902**	-0.9342**	-0.9499**	-0.9246**	-0.8723*	-0.8441*
	48h	-0.9669**	-0.9440**	-0.9161*	-0.9323**	-0.9135*	-0.8891*
2021	12h	-0.9226*	-0.9510*	-0.9745**	-0.8831*	-0.7485	-0.8252
	24h	-0.9752**	-0.9228*	-0.9654**	-0.6286	-0.8995*	-0.8574
	48h	-0.9606**	-0.8888*	-0.8820*	-0.9858**	-0.7769	-0.8162

Asterisks indicate significance at 0.05 (*) and 0.01 (**) level.

### 3.3 Low temperature on nitrogen metabolism of cotton plants

**Figure 7:**
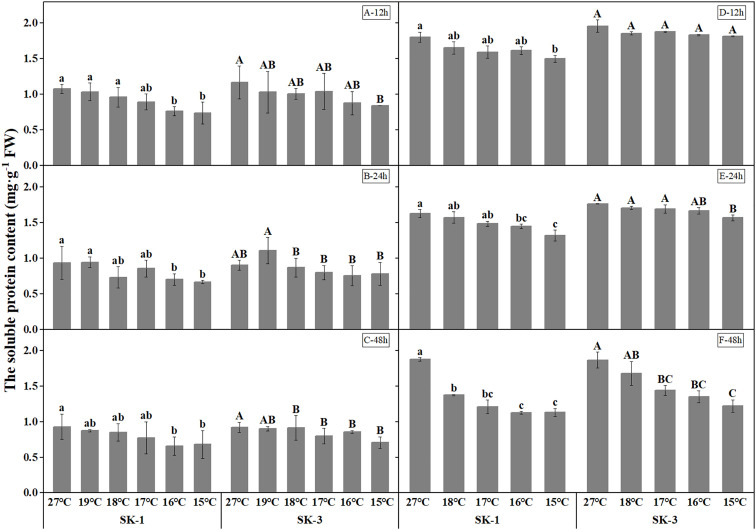
Effect of low temperature stress on soluble protein content in cotton squares in 2020 (**A-C**: 12h, 24h, 48h stress) and 2021 (**D-F**: 12h, 24h, 48h stress). FW represents fresh weight. Different lowercase letters indicate the significant differences between the treatments of the variety SK1, and different capital letters indicate the significant differences between the treatments of the variety SK3 (P<0.05).

**Figure 8:**
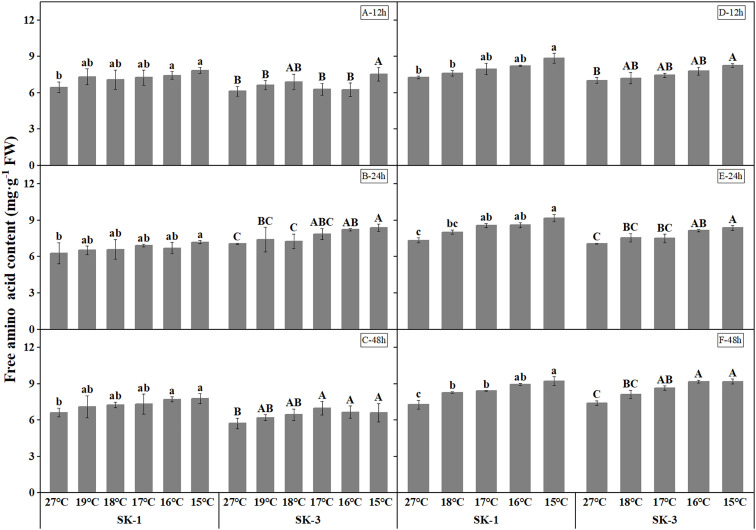
Effect of low temperature stress on free amino acid content in cotton squares in 2020 (**A-C**: 12h, 24h, 48h stress) and 2021 (**D-F**: 12h, 24h, 48h stress). FW represents fresh weight. Different lowercase letters indicate the significant differences between the treatments of the variety SK1, and different capital letters indicate the significant differences between the treatments of the variety SK3 (P<0.05).

Decrease in soluble protein contents were detected as temperature decreased (**Figure 7**). The threshold temperature causing the significant decrease of soluble protein contents in squares increased with the extension of low temperature treatment time. In 2020, the threshold low temperature was 16°C in SK1 after low temperature treatment for 12h, 24h and 48h, and 15°C and 19°C in SK3 after low temperature treatment for 12h and 48h, respectively. In 2021, similar trend was observed, with the threshold temperature as 15°C, 16°C, 18°C respectively after 12h, 24h, and 48h of low temperature stress in SK1, and 15°C and 17°C after 24h and 48h of low temperature stress in SK3. According to the above data, prolonging the treatment time of low temperature stress not only increased the critical low temperature, but also decreased the content of soluble protein in cotton buds compared with the control. In contrast to the results of soluble protein contents, free amino acid contents increased under lower treatment temperature (**Figure 8**). The threshold temperature causing the significant elevation of free amino acid contents in squares increased with the extension of low temperature treatment time. In 2020, the threshold low temperature was 16°C, 15°C, 16°C respectively in SK1 after low temperature treatment for 12h, 24h and 48h, and 15°C, 17°C, 17°C respectively in SK3. In 2021, the threshold temperature was 15°C, 17°C, 18°C respectively after 12h, 24h, and 48h of low temperature stress in SK1, and 15°C, 16°C, 17°C in SK3.

**Figure 9:**
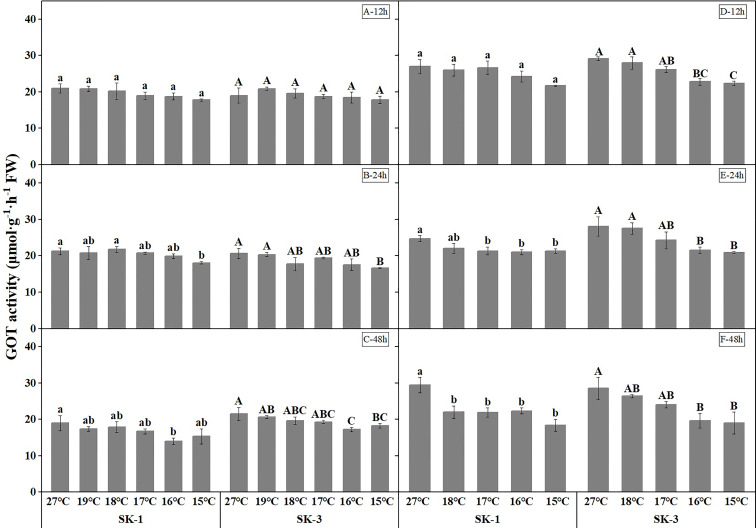
Effect of low temperature stress on GOT activity in cotton squares in 2020 (**A-C**: 12h, 24h, 48h stress) and 2021 (**D-F**: 12h, 24h, 48h stress). FW represents fresh weight. Different lowercase letters indicate the significant differences between the treatments of the variety SK1, and different capital letters indicate the significant differences between the treatments of the variety SK3 (P<0.05).

**Figure 10:**
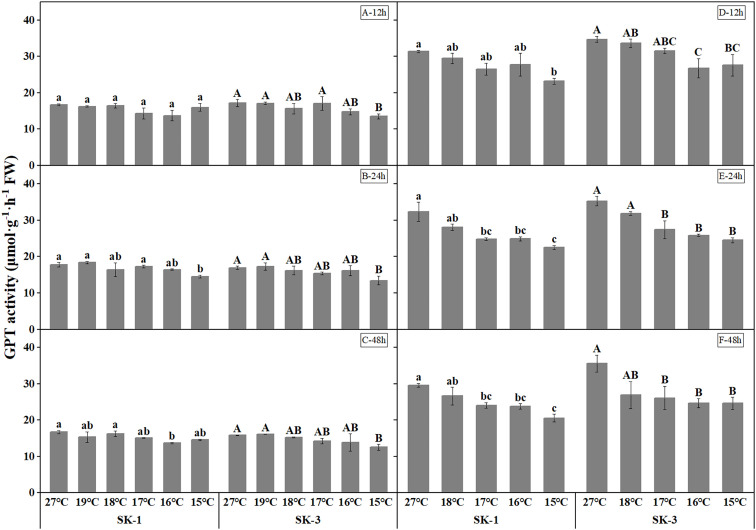
Effect of low temperature stress on GPT activity in cotton squares in 2020 (**A-C**: 12h, 24h, 48h stress) and 2021 (**D-F**: 12h, 24h, 48h stress). FW represents fresh weight. Different lowercase letters indicate the significant differences between the treatments of the variety SK1, and different capital letters indicate the significant differences between the treatments of the variety SK3 (P<0.05).

As the key enzymes in amino acid synthesis, GPT and GOT activities both decreased when temperature decreased (**Figures 9** and **Figure 10**). The threshold temperature causing the significant decrease of GPT and GOT activities in squares increased with the extension of low temperature treatment time. Prolonging the low temperature stress duration not only reduced the threshold temperature causing the significant decrease of GPT and GOT activities in squares, but also further reduced the GPT and GOT activities. Relative to the control, low temperature of 15°C decreased the GOT activity by 15.26% in SK1 and 6.22% in SK3 after 12h of stress, and the reduction of GOT activity was 14.86% in SK1 and 19.49% in SK3 after 24h of stress, and the decrease was 19.04% in SK1 and 20.15% in SK3 after 48h of stress in 2020. Similar results were observed in 2021, and the data of GPT activity showed similar trends.

**Figure 11:**
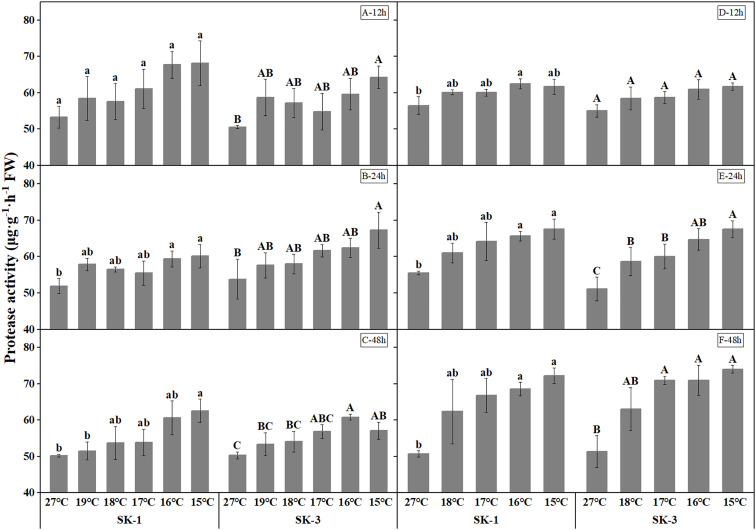
Effect of low temperature stress on protease activity in cotton squares in 2020 (**A-C**: 12h, 24h, 48h stress) and 2021 (**D-F**: 12h, 24h, 48h stress). FW represents fresh weight. Different lowercase letters indicate the significant differences between the treatments of the variety SK1, and different capital letters indicate the significant differences between the treatments of the variety SK3 (P<0.05).

**Figure 12:**
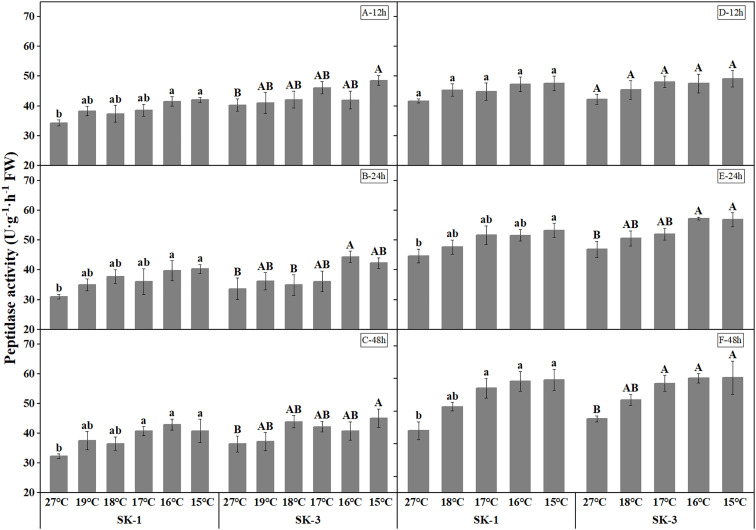
Effect of low temperature stress on peptidase activity in cotton squares in 2020 (**A-C**: 12h, 24h, 48h stress) and 2021 (**D-F**: 12h, 24h, 48h stress). FW represents fresh weight. Different lowercase letters indicate the significant differences between the treatments of the variety SK1, and different capital letters indicate the significant differences between the treatments of the variety SK3 (P<0.05).

The activities of protease and peptidase, the enzymes involved in protein decomposition, were elevated with decreasing temperature (**Figures 11** and **Figure 12**). The threshold temperature causing the significant enhancement of protease and peptidase activities in squares increased with the extension of low temperature treatment time. In 2020, the threshold low temperature for peptidase was 16°、C16°C、17°C respectively in SK1 after low temperature treatment for 12h, 24h and 48h, and 15°C、16°C、15°C respectively in SK3. In 2021, the threshold temperature was 15°C and 16°C after 24h and 48h of low temperature stress in SK1, and 16°C and 17°C after 24h and 48h of low temperature stress in SK3. Similar trends were observed in protease activity.

**Figure 13:**
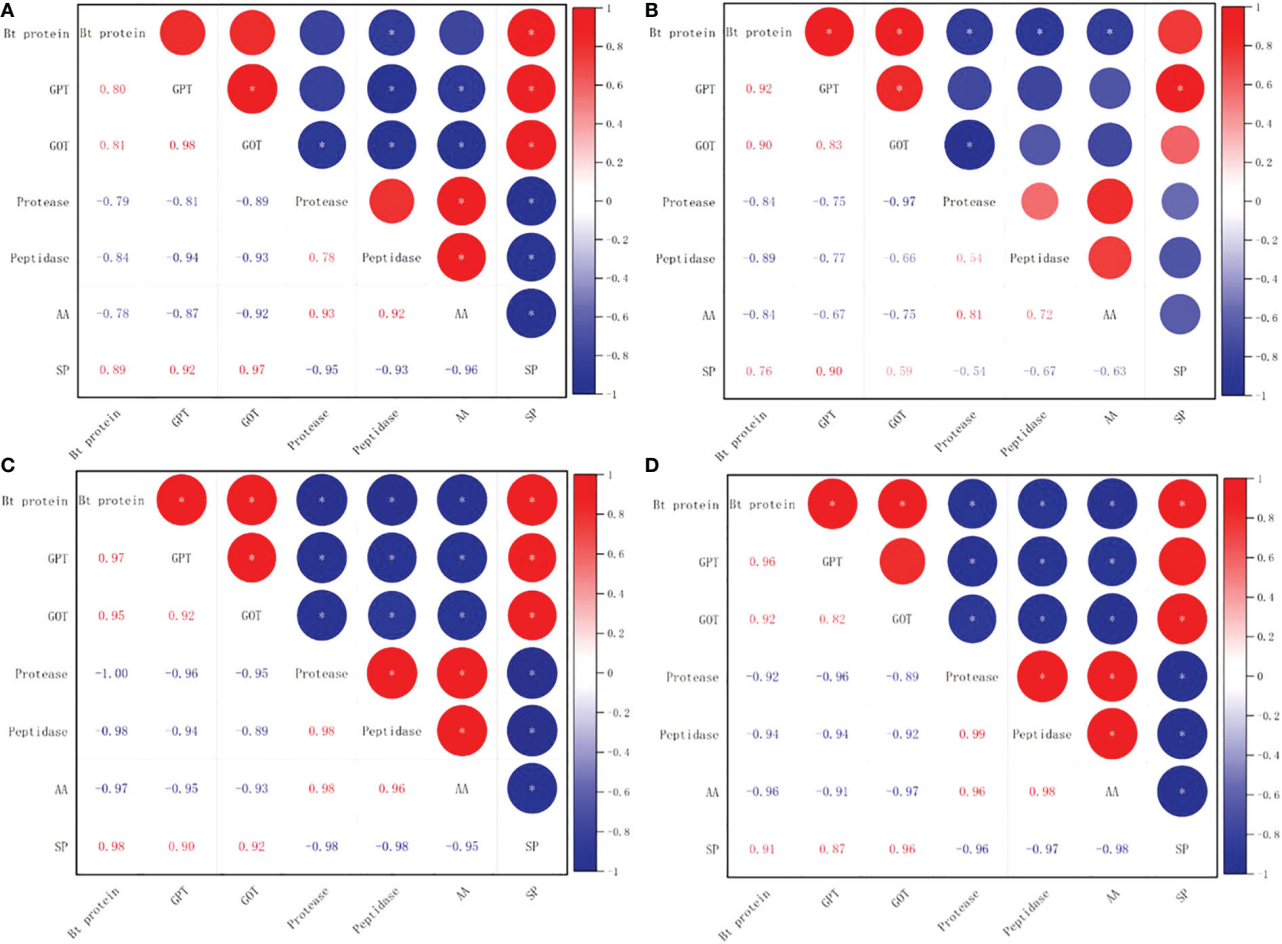
Correlations between Bt insecticidal protein content and nitrogen metabolism related parameters in 2020 (**A**:SK1, **B**:SK3) and 2021 (**C**:SK1, **D**:SK3). GPT, Glutamic-pyruvic transaminase; GOT, glutamate oxaloacetate transaminase; AA, free amino acid; SP, soluble protein. Asterisks indicate significance at the 0.05 (*) level.

There was a significant positive correlation between soluble protein level and square Bt toxin level, with values of 0.89* for SK1 and 0.76** for SK3 in 2020, and 0.98* for SK1 and 0.91* for SK3 in 2021. In addition, square Bt toxin level was significantly positively correlated with activities of GPT and GOT, but negatively correlated with free amino acid level, as well as activities of protease and peptidase (**Figure 13**).

## 4 Discussion

### 4.1 The insecticidal protein contents decreased under low temperature and squares were more sensitive compared to leaves

Zhou et al. (2015) found that the resistance of young leaves of Bt cotton to cotton bollworms significantly decreased at peak flowering and boll stage under low temperature of 16°C. Zhang et al. pointed out that low temperature stress can significantly inhibit the expression of insecticidal proteins in leaves, especially at peak boll stage (Zhang et al., 2012). The effect of temperature on insecticidal protein contents in cotton leaves was greater than that of humidity, and the effect of low temperature was greater than that of high temperature, and the insecticidal protein content of Bt cotton leaves in boll-setting stage decreased faster than that in peak flowering stage (Chen et al., 2012). However, previous researches on the effect of low temperature stress on cotton focused on leaves, and there are few studies on the effect of different low temperature levels and durations on the Bt protein contents, especially on reproductive organs. The present study indicated that the insecticidal protein contents in squares of Bt cotton decreased significantly under low temperature stress, and the threshold temperature causing a significant reduction showed an upward trend with the prolongation of stress duration, together with a greater reduction observed. In conclusion, low temperature treatment had a significant detrimental effect on the expression of insecticidal protein in cotton squares, and the threshold temperature for significantly reducing the insecticidal protein contents in cotton squares of Bt cotton was 15°C after 24 h of low temperature and 17°C after 48 h of low temperature. Therefore, in Bt cotton production, it is necessary to be alert to low temperature disasters that may last for more than 24 hours and lower than 15-17°C during the square stage, which may lead to large-scale outbreaks of pests causing serious economic losses.

Spatially, the insect resistance of different parts and organs is different, and the most important characteristic is that the insect resistance of reproductive organs is significantly lower than that of leaves (Dong and Li, 2007; Huang et al., 2010), and Bt protein content was greatest in leaves, followed by flowers, squares, and bolls, which was consistent with our results that the Bt protein contents in leaves was greater than that in squares. Across the whole treatment duration, there was a significant positive correlation between square Bt toxin level and leave Bt toxin level. Compared to the changes in square under low temperature, the insecticidal protein content in leaves showed less reduction, indicating squares were more sensitive to cold stress in terms of insecticidal protein content.

### 4.2 Low temperature induced oxidative stress of Bt cotton and thus decreased Bt protein contents

Exposing plants to temperature stress causes overproduction of reactive oxygen species (ROS), leading to oxidative damage, and exacerbating cell membrane lipid peroxidation and thus MDA production. Plants have evolved many mechanisms to prevent ROS from reaching toxic concentrations (Ashraf, 2009). Many other reports have also confirmed that under low-temperature stress, plants stimulate enzymes to alleviate oxidative damage caused by low temperature, thereby enhancing stress tolerance (Miyake and Yokota, 2000; Guo et al., 2006; Park et al., 2006; Xia et al., 2019). In our present study, the low temperature stress increased CAT and SOD activities, as well as MDA content in stressed cotton plants, and the effect of stress strengthened with lower temperature and longer stress duration.

Reduced protein synthesis was reported in stress conditions (Finkelstein and Somerville, 1990). In our present study, both soluble protein content and Bt protein contents was lower under low temperature. Across the whole treatment duration in each year, square Bt toxin level was significantly negatively correlated with MDA contents, as well as the activities of CAT and SOD, indicating the negative effect of cold induced oxidant damage on Bt protein contents. Thus, it is possible that cotton plants exposed to stress exhibited reduced protein synthesis ability and therefore decreased protein contents.

Thus, future studies that aimed to alleviate the oxidative damage caused by low temperature stress is necessary. Previous studies have shown that some exogenous substances play an important role in stress resistance. Appropriate concentration of CaCl_2_ Treatment can significantly reduce the chilling injury index, leaf relative conductivity and MDA accumulation of cotton seedlings, improve the activity of antioxidant enzymes and the content of osmoregulation substances, and effectively reduce the damage of low temperature stress to cotton seedlings (Chen and Wang, 2004). In cotton production, chitosan can protect the membrane system under low temperature stress and improve its low temperature tolerance by increasing the activity of antioxidant enzymes and the content of soluble sugar, soluble protein and proline (Xin, 2015). Under low temperature conditions, exogenous NO can resist the damage of cell membrane peroxidation by promoting the activity and content of SOD, POD and CAT antioxidant enzymes, and thus alleviate the damage of cold stress (Li et al., 2011). Melatonin could increase the activity of SOD, POD, and GA content, and reduce the content of MDA and ABA in cotton under drought or salt stress conditions (Xiao et al., 2019). Therefore, the application of exogenous substances may be an effective method to improve the cold tolerance of crops, and their effect on Bt protein contents need to be explored in future.

### 4.3 Low temperature altered nitrogen metabolism and thus decreased Bt protein contents

Chen et al. found that the expression of insecticidal proteins in cotton leaves is closely related to nitrogen metabolism (Chen et al., 2004; Pettigrew and Adamczyk, 2006; Luo et al., 2008; Chen et al., 2018; Chen et al., 2019). This study found that the soluble protein content, GOT and GPT activities decreased under low temperature stress, while the free amino acid content, peptidase and protease activities increased under low temperature stress in cotton squares, with greater change was noted as the treatment temperature decreased. The above results show the reduction of insecticidal protein contents in cotton squares under low temperature was a combined effect of weakened protein synthesis ability and enhanced protein decomposition ability. Therefore, in square stage that is prone to low temperature injury, it is necessary to pay attention to the change of the insecticidal protein content of Bt cotton, take corresponding measures in time to increase nitrogen metabolism and thus reducing the negative impact of low temperature and chilling injury on cotton.

## 5 Conclusions

The insecticidal protein contents in squares and leaves of Bt cotton decreased significantly under low temperature stress, and the threshold temperature causing a significant reduction showed an upward trend with the prolongation of stress duration, together with a greater reduction observed. Under low temperature, reduced MDA contents, as well as the activities of CAT and SOD was detected, indicating the cold induced oxidative damage was associated with Bt protein contents. Besides the oxidative stress under low temperature, the analysis of N metabolism showed that soluble protein content, GPT and GOT activities decreased, free amino acid, peptidase and protease activities increased under low temperature. Thus, enhance protein decomposition and reduced protein synthesis functioned together to decrease Bt protein contents under low temperature.

## Data availability statement

The original contributions presented in the study are included in the article/supplementary material. Further inquiries can be directed to the corresponding author.

## Author contributions

YC and ZL conceived the study. XZ and DC supervised the study. ZL, YD, YY, YL, and HL performed the experiments. YC, ZL, and RH analyzed the data. YC and ZL drafted the manuscript. YC and ZL revised the manuscript. All authors contributed to the article and approved the submitted version.
